# An Owner-Independent Investigation of Diabetes Alert Dog Performance

**DOI:** 10.3389/fvets.2019.00091

**Published:** 2019-03-27

**Authors:** Clara Wilson, Steve Morant, Sarah Kane, Claire Pesterfield, Claire Guest, Nicola J. Rooney

**Affiliations:** ^1^Animal Welfare and Behaviour Group, Bristol Veterinary School, Bristol, United Kingdom; ^2^Medicines Monitoring Unit (MEMO), School of Medicine, The University of Dundee, Dundee, United Kingdom; ^3^The Department of Biology, Hamilton College, Clinton, NY, United States; ^4^Medical Detection Dogs, Milton Keynes, United Kingdom

**Keywords:** hypoglycaemia, hyperglycaemia, diabetes, alert, canine, behavior

## Abstract

**Objective:** To quantify Diabetes Alert Dog (DAD) performance by using owner-independent measures.

**Research Design and Methods:** Eight owners of accredited DADs used a FreeStyle Libre Flash Glucose Monitoring System (FGMS). Concurrent Closed Circuit Television (CCTV) footage was collected for between 5 and 14 days in each owner's home or workplace. The footage was blind-coded for dogs' alerting behaviors. The sensitivity, False Positive Rate and Positive Predictive Values (PPV) of dogs' alerts to out-of-range (OOR) episodes were calculated. Ratings for 11 attributes describing participant's lifestyle and compliance (taken from each dog's instructor) and the percentage of DAD alerts responded to by the owner as per training protocol (taken from CCTV footage) were assessed for association with dog performance.

**Results:** Dogs alerted more often when their owners' glucose levels were outside *vs*. inside target range (hypoglycaemic 2.80-fold, *p* = 0.001; hyperglycaemic 2.29-fold, *p* = 0.005). Sensitivity to hypoglycaemic episodes ranged from 33.3 to 91.7%, the mean was 55.9%. Mean PPV for OOR episodes was 69.7%. Sensitivity and PPV were associated with aspects of the dog and owner's behavior, and the owner's adherence to training protocol.

**Conclusions:** Owner-independent methods support that some dogs alert to hypo- and hyperglycaemic events accurately, but performance varies between dogs. We find that DAD performance is affected by traits and behaviors of both the dog and owner. Combined with existing research showing the perceived psychosocial value and reduced critical health care needs of DAD users, this study supports the value of a DAD as part of a diabetes care plan. It also highlights the importance of ongoing training and continued monitoring to ensure optimal performance.

## Introduction

There are an estimated 4.6 million people in the United Kingdom living with diabetes (1). Of those, ~400,000 are currently living with Type 1 diabetes, the incidence of which is increasing by around 4% each year (2). Without extraneous insulin intervention, blood glucose levels are susceptible to becoming too high (hyperglycaemia) or too low (hypoglycaemia). This results from a failure of the body to produce insulin, and people with Type 1 diabetes must utilize exogenous insulin via regular injections or a continuous infusion to maintain blood glucose levels within a “prescribed target range” in order to limit the risk of developing long term complications associated with this condition (3). Whilst there exists clinical definitions of hypo- and hyperglycaemia, many individuals living with Type 1 diabetes will use approximations of these values as they may experience physiological symptoms of hypo- and hyperglycaemia at different blood glucose levels. Each person's “target range” is clinician-guided and based on personal experience at certain “low” and “high” blood glucose values, with measures inside of this range deemed safe for that individual. Outside of this range, corrective measures are required (1). Hypoglycaemia is a prevalent and serious complication of diabetes. Mild episodes can interfere with everyday functioning, while a severe episode requires intervention from another person and, if left untreated, can be fatal (4, 5). People with Type 1 diabetes can become unaware of the symptoms of hypoglycaemia over time, which has been found to increase the risk of a severe hypoglycaemic episode six to seven-fold (6), and is associated with an increased risk of mortality (7). Fear of hypoglycaemia causes some people to restrict their lifestyle in efforts to reduce the likelihood of an episode, which negatively impacts both their quality of life and psychological well-being (8). Individuals may intentionally “run their blood sugars high” (maintaining hyperglycaemia) because of fear of a severe hypoglycaemic episode (9). This practice confers various associated health risks over time, such as cardiovascular disease, nerve, and kidney damage (10, 11).

While an array of developing technologies are available to people with Type 1 diabetes, many are invasive, requiring either finger-pricks or sensor insertion, and can carry considerable financial burdens (e.g., sensor replacement) or physical equipment (12). Diabetes Alert Dogs (DADs) potentially offer a non-invasive method of assisting in the recognition of an oncoming hypo- or hyperglycaemic episode by alerting while their owner is still able to act (13), a concept that has led to an increase in popularity over the last decade in using DADs as a method to facilitate tightened glycemic control (14). DADs are trained to alert their owner by performing attention-gaining behaviors when glucose levels deviate from their target range. Their potential benefits are substantial, preventing patients with glycemic unawareness experiencing dangerous glucose fluctuations, thereby improving owners' quality of life and potentially reducing mortality rates (15, 16). Given the health risks associated with diabetes, it is imperative that the efficacy and value of DADs are objectively assessed.

There have been 22 previous studies on DADs: seven are owner-informed case reports of untrained and trained dogs (13, 17–22), five use *in vitro* laboratory testing (23–26) and the remaining ten use owner-reported information for at least one aspect of data collection (12, 15, 16, 27–32). As Weber et al.'s (33) review highlights, small sample sizes and inconsistent sampling methods make drawing confident conclusions problematic. Prior to this study, there have been no entirely owner-independent assessments of *in-situ* DAD performance.

Rooney et al.'s (32) study of 27 DADs suggests that the accuracy of some dogs is very high, with a median sensitivity to hypoglycaemic episodes of 83%. This is currently the largest single agency study, however, it relies upon owner reports of DAD alerts and owner provided blood-test data. This could result in undetected false negatives; when owners are unaware that their blood glucose has fluctuated outside of their target range and their DAD has failed to alert them (however, this may only impact the number of mild episodes recorded as a severe hypoglycaemic event is likely to be recognized due to physiological effects). Therefore, reported sensitivity of DAD alerts in studies that use point-in-time blood test results [e.g., (12, 16)] may be artificially high, as fluctuation into hypo- or hyperglycaemia that did not produce a noticeable physiological effect and to which a DAD did not alert may have been unreported. Using a monitor that records glucose levels at regular intervals to establish periods of euglycaemia and hypo-/hyperglycaemia is therefore integral to accurately assess DAD alerting sensitivity rates. Furthermore, owners may fail to accurately record false positives (alerts occurring during in-range glucose levels), thus previously reported positive predictive values (PPV) of dog alerts could also be artificially high.

Two recent experimental studies overcame the issue of potentially missing false negatives by utilizing Continuous Glucose Monitoring Systems (CGMS) (30, 31). CGMS are owner-independent as they automatically record interstitial fluid glucose levels via a sensor inserted under the skin, which facilitates a more accurate measure of DAD sensitivity since all OOR episodes are recorded. These recent studies however still rely upon owner reports of DAD alerts. Los et al. (30) found that a cohort of eight DADs from multiple training backgrounds performed variably, with an average sensitivity of 36% to hypoglycaemic events and a PPV of only 12%. However, seven of the eight dogs sampled had been trained to alert to hyperglycaemia, yet only alerts to hypoglycaemia were considered correct. Hence, of the reported 88% “incorrect” alerts, it is unknown what proportion were actually events where the dog was alerting to hyperglycaemia. Gonder-Frederick et al. (31) collected CGMS data, blood test readings, and owner reports of DAD alerts from 14 participants over 6 weeks, and similarly found substantial variation in performance between dogs, with only three out of 14 dogs performing statistically above chance level. The cause of this variability is as yet unexplored.

Whether a dog's alert is considered “correct” will depend on the glucose values used to determine hypo- and hyperglycaemia. Both Los et al. (30) and Gonder-Frederick et al. (31) used the clinical definition of glycaemic states (≤ 3.9 mmol/L: hypoglycaemia and ≥10.0 mmol/L: hyperglycaemia) whilst Gonder-Frederick et al. (31) also considered “more extreme” hypoglycaemic (≤ 3.0 mmol/L) and hyperglycaemic states (≥13.9–16.7 mmol/L). Many dogs are trained to respond to their individual owner's target glucose range (32), so testing their accuracy using these ranges may give a fairer assessment of efficacy, whilst considering extreme glucose levels gives an indication of their value at preventing severe episodes and requiring paramedic call outs. Gonder-Frederick et al. (31) saw no substantive differences in accuracy to “extreme” glucose levels as compared to the standard definitions of hypo- and hyperglycaemia, however, no previous study has assessed accuracy levels to owners' *individual* target ranges and extreme glucose levels.

The current study was conducted using dogs trained by Medical Detection Dogs (MDD), the only training institution for DADs in the United Kingdom accredited by Assistance Dogs UK. Initial training uses *in vitro* samples obtained from the dog's prospective owner when they are in a hypoglycaemic state, paired with a reward to shape the dog's response to the odor. Response behaviors, which are reinforced, include staring, pawing, licking the owner and/or fetching the owner's blood testing kit (34). *In vitro* training continues for ~7 weeks. Once a dog is deemed to be consistently responding to these samples in a variety of environments they are placed with their prospective owner and taught to alert in response to the client's target glucose range. Ongoing support and regular assessments of sensitivity and accuracy of the dog's alerting behavior provide information of the success of the transfer from *in vitro* training to *in vivo* alerting. When dogs alert, owners should confirm if they are correct using a blood test. Only if glucose levels are confirmed as outside of their target range should they reward their dog. During training at MDD, dogs are presented with hypoglycaemic samples only, since reducing the likelihood of a life-threatening hypoglycaemic event is the primary aim. However, most dogs subsequently develop spontaneous alerts to hyperglycaemia, which owners are advised to reward (with a lower value reward). Once accredited, the owner is responsible for rewarding their dog and hence maintaining its performance, however systematic instructor visits are carried out. Clients also provide dog alerting and blood test data annually to allow performance to be monitored and re-accredited annually in accordance with Assistance Dog International guidelines.

FGMS and CGMS are currently the best devices with which to compare DAD performance due to their objectivity and facilitation of recording many data points over a period of time [e.g., (30, 31)]. These are devices that use sensors beneath the skin to sample interstitial fluid, in the case of CGMS, continually, or for FGMS at regular intervals, displayed to the user when they choose to scan the sensor. However, DAD behavior is shaped using the results of finger-prick blood tests, and readings taken by each device often differ even when taken at the same time point (35, 36), which may impact upon measured DAD performance. The degree of agreement between blood tests and FGMS readings can be quantified using Clarke-Error Grids (37) and likely varies between individuals (38, 39). Here we explore its association to measured DAD performance.

DAD owners anecdotally report that dogs alert to oncoming episodes before their glucose has fallen outside their target value (32). By using a Glucose Monitoring System, which provides estimates of the time of *transition* from in-range to OOR, we can further explore evidence of pre-alerting. Furthermore, owners report that they may reward their dog for values approximate to their specified low or high glucose value. Here we additionally assess the impact of a fixed vs. ± 0.5 mmol/L margin of error for which values are considered “correct” when assessing DAD performance.

Lastly, an understanding of factors leading to variation in DAD accuracy is vital in order to improve performance in the future. Rooney et al. (32) suggest that behavioral traits in dogs, as well as owners, may contribute. In particular, each owner's response to alerting behavior, and the impact of following the recommended reward regime instilled during training, is likely to be important. This has not been possible to test directly in previous studies due to the reliance on owner reports of DAD alerts. Here, utilizing CCTV footage, we can for the first time assess whether owner compliance may have an effect on DAD performance.

The current study presents the first entirely owner-independent assessment of *in-situ* DAD accuracy. We use FGMS to record owner blood glucose levels and CCTV cameras to assess DAD and owner behavior, to address five questions:

1. Do dogs accurately alert their owners to hypo- and hyperglycaemic episodes as identified by interstitial glucose monitoring?
2. Does using individual glucose target ranges, as compared to the clinical definition of extreme hypo- and hyperglycaemia, affect calculated alert accuracy?
3. Does variation in analysis approach alter reported rates of DAD accuracy? Specifically, does including a 15-min window prior the first OOR FGMS reading (which may include evidence of pre-alerting), or including a ± 0.5 mmol/L range around owners specified glucose target values, alter calculated DAD performance?
4. How do clients' FGMS values compare to blood test results, and does the level of agreement affect their DAD's measured performance?
5. Are aspects of owner lifestyle, compliance and behavior associated with dogs' alerting accuracy?

Elsewhere we report in detail the same cohort's objective behaviors during pre-defined periods of owner glucose stability or fluctuation (40). Here we report accuracy of DAD alerts and factors that may affect it.

## Research Design and Methods

### Recruitment

Individuals were approached via telephone if they had previously expressed an interest in taking part in research, owned an accredited DAD trained by Medical Detection Dogs, and were above the age of 18. Of the 14 approached, nine accepted. Participants were sent information via email detailing the study protocol and reminding them of their right to withdraw; however none did. One dog was subsequently found to alert their owner using vocalizations as well as motor behaviors, and since the project relies on silent video footage, this dog was removed from analysis since some vocal alerts may have been missed.

### Participants

Participants were seven female and one male with Type 1 diabetes, ranging from 26 to 63 years (*Median* = 52.2 years). Dogs were six neutered males and two spayed females. Breeds included four Labradors, two Labrador-Golden Retriever crosses, one Miniature Poodle and one Golden Retriever. All pairs had been accredited between 12 and 72 months (*Median* = 47.1 months).

### Initial Visit

Participants were visited in their home (seven) or place of work (one). They were instructed to continue their pre-existing diabetes management without alteration and asked to provide their blood test results for the duration of the study. Their target blood glucose range was recorded (Table 1). Participants were provided with an information sheet, consent form, video record sheet (to indicate periods of footage not to be viewed), blood test record sheet and FGMS instruction sheet.

**Table 1:** Information on footage collected during the study period for each participant and each individual's target glucose range.

**Number of cameras installed**	**Total hours of in-sight footage collected**	**Low glucose value < (mmol/L)**	**High glucose value > (mmol/L)**
3	130	5.0	12.0
2	42	4.0	10.0
3	87	4.5	14.0
2	81	6.0	10.0
4	60	5.0	15.0
3	101	4.7	11.0
2	116	4.0	15.0
3	77	4.5	10.0

*Values above or below these parameters are considered “out-of-range” for that individual. Each line refers to one participant. Participant numbers have been removed for anonymity*.

### Flash Glucose Monitoring System

Participants were each loaned a FreeStyle Libre Flash Glucose Monitoring scanner (Abbott Diabetes Care, Almeda, CA) and were assisted to insert a sensor to be worn for 14 days (after which the sensor expires). Three participants did not complete the full study period, two for personal reasons (after five and 10 days respectively) and one because the sensor fell off after 13 days. A 6 × 6 cm opaque plastic square was placed over the scanner screen to occlude glucose level results, mitigating the risk of participants modifying their behavior in response to on-screen glucose levels. The FGMS device logs glucose values every 15 min, and stores additional data points every time the sensor is scanned.

### Cameras

Swann CCTV Systems were used, with footage stored on a Swann Digital Video Recorder (DVR). Between two and four cameras were mounted in the rooms in which the participant reported spending most of their time, maximizing the time in view. Four participants allowed footage to be taken in their bedroom during sleeping hours to capture nocturnal alerts. Total hours of footage collected with owner and DAD in-view ranged from 42 to 130 h (Mean = 86.8 h) (Table 1).

### Data Collation

FGMS values were uploaded at The University of Bristol using the FreeStyle Libre software (version 1.0). Each FGMS data point was categorized as “hypoglycaemic,” “in-range,” or “hyperglycaemic” depending on the individual's target range. In subsequent analysis this procedure was repeated using the clinical definition of “severe” hypoglycaemia (3.0 mmol/L) and hyperglycaemia (13.9 mmol/L) to categorize OOR episodes (31, 41, 42).

### Video Data

For each dog, behaviors that constituted an alert were established from their instructor (see Table 2). Behavioral coding was carried out using The Nodulus Observer XT Version 11.5. The footage was watched in real time and the frequency of alerts was recorded. For each alert, the time of occurrence, owner's response (including whether the owner tested their blood, whether they rewarded the dog and, if so, whether it was before or after the blood test) and whether the dog's alerting behavior was deemed unambiguous or ambiguous was also recorded. Researchers were blinded to the FGMS values when observing the footage, which included no sound for participant privacy and to ameliorate bias. Participant Two and Seven's footage was second-coded to establish inter-rater reliability.

**Table 2:** Alerting behaviors shown by each dog.

		**Partnership**							
**Behavior**	**Definition**	**1**	**2**	**3**	**4**	**5**	**6**	**7**	**8**
Fetch blood testing kit or treatment in its mouth	Dog picks up the blood testing kit or energy drink bottle in its mouth and approaches the owner.	X		X	X	X	X	X	
Stare at owner	Dog shows fixed eye contact toward owner with eyes wide open.	X	X	X	X				X
Nuzzle owner	Dog pushes face into any part of the owner's body or clothes. Must be in contact with owner.		X				X	X	
Mouth owner	Dog manipulates any part of owner's body to be held in their jaw. Must be in contact with owner.						X	X	
Paw owner	Dog lifts one front foot to make contact with the owner.	X	X			X	X	X	
Lick owner	Dog makes contact with any part of the owner's body using its tongue.						X		X
Jump up on owner	Dog lifts both front paws, or all four paws off the ground and makes contact with the owner.	X	X	X					

*Shaded boxes indicate behaviors identified by the DAD's instructor as elicited when alerting*.

### Statistical Methods

#### Inter-rater Reliability

The number of video segments in which alerts were and were not recorded by each coder were tabulated and compared using Cohen's Kappa.

#### In-range and Out-of-Range (OOR) Episodes

OOR episodes were defined by sets of consecutive interstitial glucose readings beyond each participant's limits for hypo- or hyperglycaemia. The beginning and end-point of each episode were estimated by linear interpolation between the first OOR reading and the previous reading, and the last OOR reading and the next reading.

#### Alert Rates

The total lengths of each participant's in-range, hypo- and hyperglycaemic episodes, and the number of alerts that occurred within each were calculated. The rates of alerts during periods that were OOR to those during in-range episodes were compared using a generalized linear model with Poisson errors. Given that euglycaemia forms continuous rather than discrete events an appropriate denominator cannot be defined for specificity, hence we calculated False Positive Rates (FPR) and Positive Predictive Values (PPV) instead. The rates of alerts that occurred during in-range periods formed the FPR. The generalized linear models used either a log link function (Poisson data) or logit link function (binomial data), and included a scale parameter to account for over dispersion between dogs.

#### Sensitivity

Sensitivity was calculated as the proportion of OOR episodes with at least one alert within 15 min prior to the beginning of the OOR episode and the end of the episode. Episodes where the dog was out of sight for two or more of the automatic glucose readings were excluded. In a supplementary analysis, alerts in the 15 min prior to the start of an OOR episode were excluded to assess whether excluding pre-alerts impacted on performance estimates. Exact confidence intervals were calculated for the sensitivity of each dog. A generalized linear model with binomial errors was used to estimate the confidence interval for sensitivity averaged over all dogs.

#### Positive Predictive Value (PPV)

PPV was calculated as the proportion of observed alerts that occurred during, or up to 15 min prior to, an OOR episode. Exact confidence intervals were calculated for each dog. A generalized linear model with binomial errors was used to estimate the confidence interval for PPV averaged over all dogs. A supplementary analysis using clinical definitions of “extreme” hypo- and hyperglycaemic events using a glucose value of ≤3.0 mmol/L for hypoglycaemia and ≥13.9 mmol/L for hyperglycaemia was carried out. Since owners reported that they sometimes reward their dog on occasions where their glucose value was approaching their target value (rather than using the exact value), we repeated the analysis with a ± 0.5 mmol/L margin of error.

#### Clarke Error Grids

Interstitial glucose vs. blood glucose were plotted for each partnership using the FGMS reading recorded closest in time to each blood sample and Clarke Error Grids were constructed (37). Results in zones A and B are considered clinically acceptable [(35) c.f. (43)]. The FreeStyle Libre system is reported to have an accuracy of 99.7% of data points within zones A and B (35).

#### Instructor Ratings

The individual who had trained each DAD partnership was provided with a questionnaire rating 11 attributes taken from Rooney et al. (32) where instructor interviews were used to identify factors deemed important to the training process. They were: *Busyness of the Household, Severity of Client's Diabetes, Speed of Client's Glucose Drops, Client's Willingness to Reward the Alerts, Client's Ability to Recognize the Dog's Alerts, Client's Confidence in the Dog's Ability, Consistency of Client's Behaviour Towards Dog, Client's Level of Communication with Instructor, Dog's Motivation and Enjoyment of the Task, Strength of Dog's Alert, Dog's Willingness to Try New Behaviours and “Get it Wrong*.” All of these attributes were rated 1 (Very low) to 10 (Very high). In addition, time since accreditation (months); Number of people in household; and Children in the household (Yes/No) were collected. Generalized linear models with binomial errors were used to assess the value of these scores as predictors of sensitivity and PPV. The percentage of alerts followed by a blood test, the percentage of alerts ignored by owner (as taken from the CCTV footage), and the percentage of FGMS results in zones A and B were also assessed as potential predictors.

Across all analyses, response alerts (those that occur immediately after the owner conducts a blood test) and ambiguous alerts were excluded. SAS V9.4 was used for all statistical analyses.

## Results

There was a strong agreement between the two observers' judgment for Participant Two (*K* = 0.85, 95% CI, 0.73, 0.97, *p* < 0.0001) and a moderate agreement for Participant Seven (*K* = 0.70, 95% CI, 0.59, 0.82, *p* < 0.0001) (44).

Do dogs accurately alert their owners to both low and high glucose episodes?

All dogs alerted more frequently during hypoglycaemic episodes than during in-range episodes, on average by a factor of 2.80 (95% CI 1.67, 4.68; *p* < 0.001). Six of the eight dogs alerted more frequently during hyperglycaemic episodes, on average by a factor of 2.29 (95% CI 1.29, 4.05; *p* = 0.005). Overall relative rate of alerts occurring during in-range periods (False Positive Rate) was 0.19 per hour.

### Sensitivity

**Figure 1:**
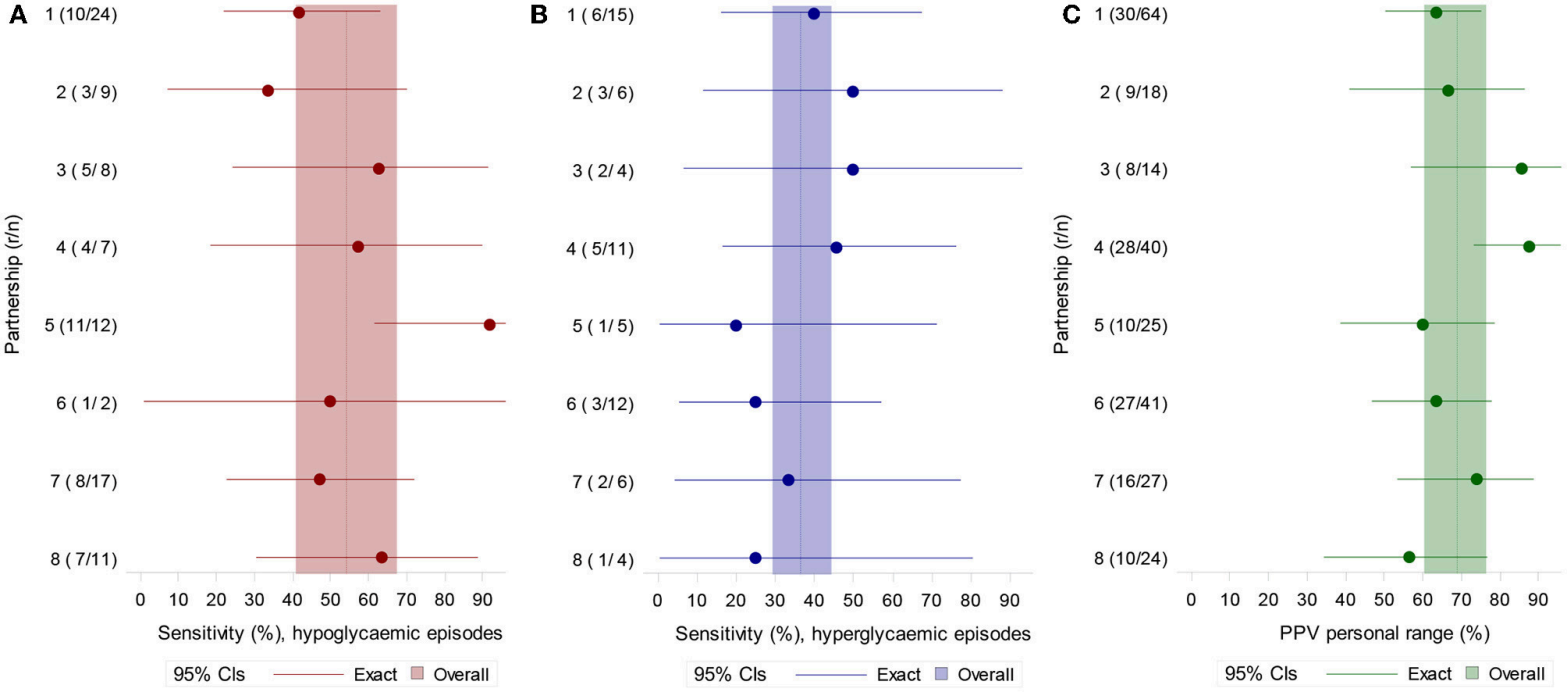
**(A)** Sensitivity for hypoglycaemic (left) and **(B)** hyperglycaemic episodes (middle) defined by each participant's own target range. **(C)** PPV for OOR episodes defined by each participant's target range (right). *N* = number of episodes, *r* = episodes with at least one alert during the episode or in the 15 min preceding it.

Ninety hypoglycaemic episodes and 63 hyperglycaemic episodes were identified in the eight participants, defined by their individual target ranges. Sensitivity to hypoglycaemic episodes overall was 55.9% (95% CI 40.8, 67.4) with individual dogs ranging from 33.3 to 91.7% (Figure 1A). When using the definition of severe hypoglycaemia (3 mmol/L), the mean sensitivity was similar: 54.2% overall (95% CI 37.6, 70.0). Sensitivity to hyperglycaemic episodes was 36.5% (95% CI 29.3, 44.4) (Figure 1B).

**Figure 2:**
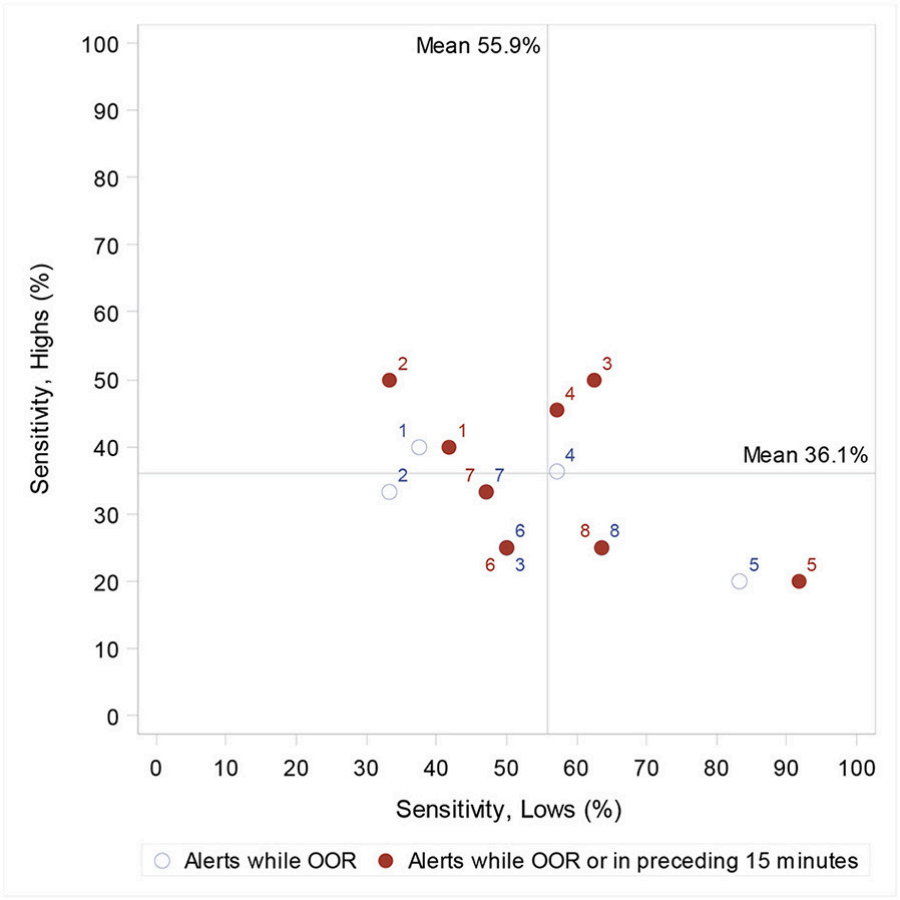
Individual dog's sensitivity to highs (hyperglycaemia) and lows (hypoglycaemia), and, when considering only those alerts once the owner is OOR, and when also including alerts 15 min prior to the first OOR recording. When two circles (one closed and one open) have the same dog number, this indicates a change in sensitivity when alerts in the processing 15 min are considered correct. Lines indicate the population mean (when including the preceding 15 min).

When alerts in the 15-min period prior to an OOR episode were considered “incorrect” (excluded), sensitivity was reduced from 55.9 to 51.1% for hypoglycaemic episodes (95% CI 39.3, 62.8) and reduced from 36.5 to 31.7% for hyperglycaemic episodes (95% CI 26.9, 37.0) (Figure 2).

### Positive Predictive Value

PPV using each participant's own target range was 69.7% overall (95% CI 60.3, 76.5) (Figure 1C). Using the definition of more extreme hypoglycaemia and hyperglycaemia, PPV was 50.4% overall (95% CI 39.4, 61.3). When OOR episodes included a ± 0.5 mmol/L margin of error, PPV to OOR episodes became 75.3% (95% CI 67.0, 82.1).

### FGMS and Blood Test Accuracy

**Figure 3:**
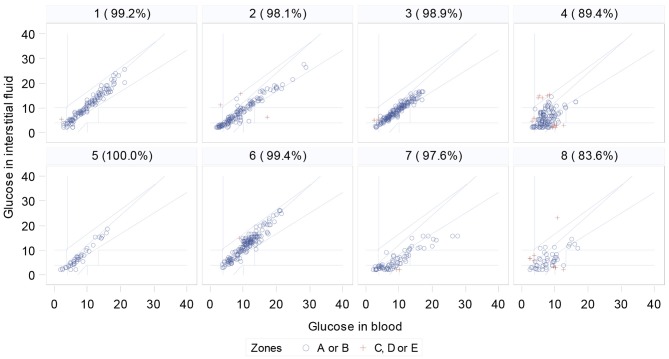
Clarke Error grids for each participant, comparing FreeStyle Libre FGMS with blood test results (% is percentage of data in zones A or B. Red crosses are data points outside these zones).

**Figure 4:**
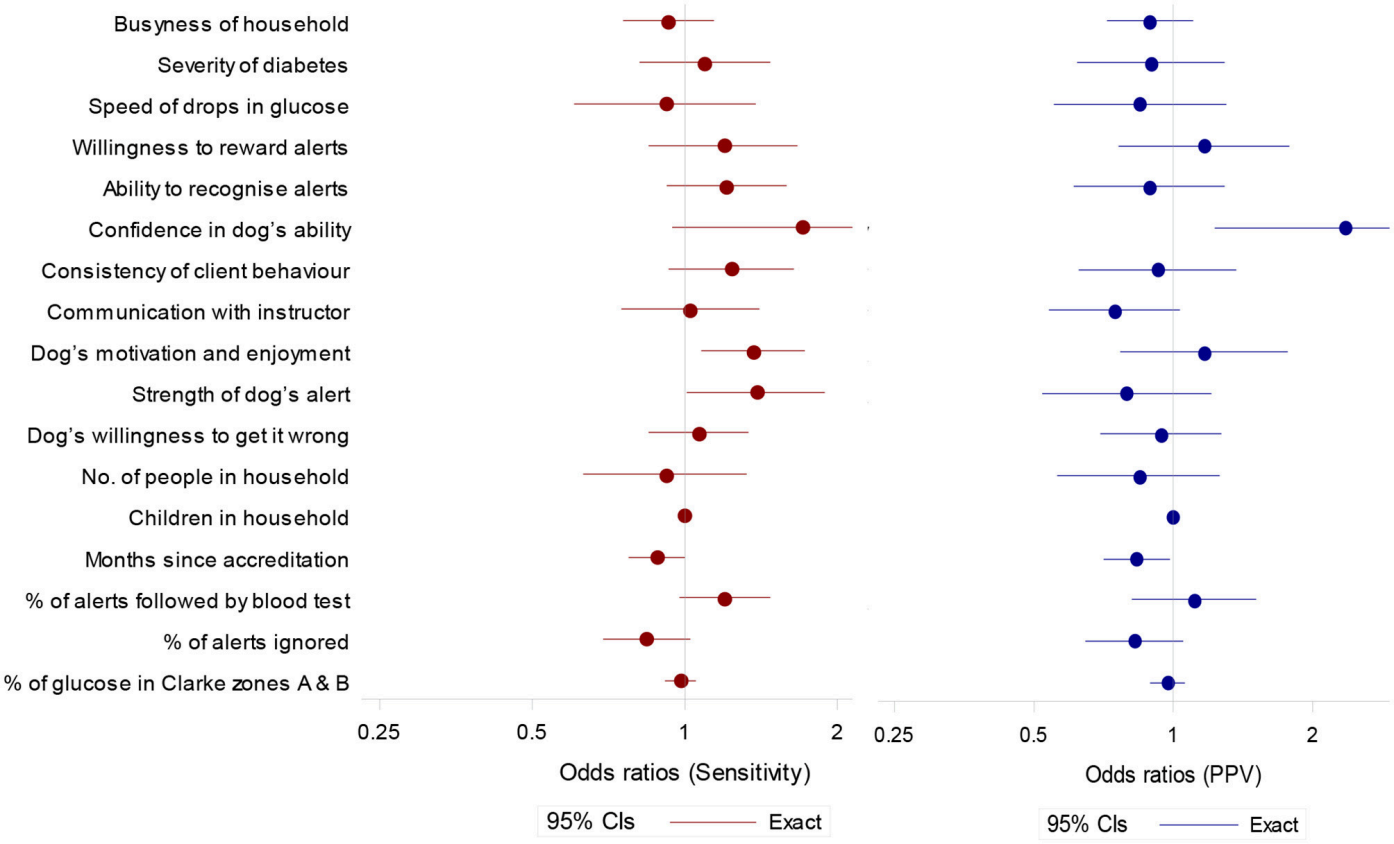
Odds ratios (95% confidence intervals) for potential predictor of sensitivity (to OOR episodes) and positive predictive value. Ratios are for a unit increase in score except for months since accreditation (per year), and % of alerts followed by blood test and percentage of alerts ignored (per 10 percentage point increase).

Clarke Error grids show that the percentage of readings in zones A and B ranged from 83.64 to 100% (Figure 3). Agreement levels between devices were not significantly associated with measured DAD performance (Figure 4).

### Owner Response to DAD alerts

Participant Two and Five showed a 100% adherence to training protocol by appropriately responding to all their DAD's alerts. Five of the eight participants ignored alerts on at least one occasion. Two of the eight participants rewarded their DAD prior to testing blood glucose levels on at least one occasion (Table 3).

**Table 3:** Owner responses to DAD spontaneous alerts, and number of response alerts.

	**Spontaneous alerts**				**Response alerts**
**Partnership**	**Number of spontaneous alerts**.	**Percentage of spontaneous alerts in response to which owner tested their blood (as per training protocol)**.	**Percentage of spontaneous alerts ignored: i.e., the owner responded with neither a blood test nor a reward. Numbers in brackets are the percentage of ignored alerts that occurred within 15 min of previous alert (repeated alerts)**.	**Percentage of spontaneous alerts that owner gives DAD reward without testing blood**.	**Number of alerts occurring immediately after owner carries out routine test**.
1	73	61.6	38.4 (9.6)	0	5
2	18	83.3	11.1	5.6	0
3	20	100	0	0	3
4	40	80	0	20	11
5	22	100	0	0	0
6	44	79.5	20.5 (20.5)	0	4
7	28	96.4	3.6	0	0
8	23	91.3	8.7	0	1

*Training protocol states that owners should respond to a spontaneous alert only after a confirmatory blood test, unless the DAD has been rewarded for a correct alert and then repeats the alert shortly after being rewarded (e.g., <15 min)*.

*Green shading denotes “correct” owner response in-line with training protocol. Red denotes owner responses not recommended in training. Blue shading denotes “response alerts” which are distinct from spontaneous alerts as they occur immediately after an owner takes a routine test and thus the alert is likely prompted by the visual cue of the owner testing their blood*.

### Instructor Ratings

Sensitivity tended to be higher in partnerships with higher scores for *Client's Confidence in the Dog's Ability, Dog's Motivation and Enjoyment of the Task, Strength of Dog's Alert* and a higher observed percentage of alerts followed by a blood test (Figure 4). Sensitivity tended to be lower if the observed percentage of alerts ignored was higher (Figure 4).

PPV tended to be higher in partnerships with a high score for *Client's Confidence in the Dog's Ability* (Figure 4). A longer length of time since accreditation was associated with lower scores for both sensitivity and PPV.

## Discussion

This study is the first to demonstrate, using objective measures *in-situ*, that dogs detect episodes of both low and high blood glucose levels. The cohort showed alerts 2.80-fold more often in hypoglycaemic episodes and 2.29-fold in hyperglycaemic episodes compared to when their owner was in-range. Some dogs performed with very high levels of sensitivity and PPV, however substantial variation was seen despite all dogs having been trained by the same institution and following the same protocol for accreditation.

Using CCTV footage and FGMS we found an overall sensitivity to hypoglycaemia of 55.9%, and to hyperglycaemia of 36.4%. PPV (proportion of alerts that were correct) averaged 69.7%. Some dogs were performing with great sensitivity (maximum of 91.7%, Dog Five) and high PPV (maximum of 87.5%, Dog Four). Sensitivity to hyperglycemia was generally lower than that to hypoglycaemia, as would be expected given that dogs are formally trained on hypoglycemic scent only. However, all eight dogs alerted to some hyperglycaemic episodes, with one dog (Dog Two) showing higher sensitivity to hyperglycaemia than to hypoglycaemia. This supports Rooney et al.'s (16) finding that DADs not only can prevent dangerous hypoglycaemic episodes but can also facilitate tighter glycemic control. Measuring intervention effectiveness in terms of hypoglycaemia only [e.g., (30)] has limited value, as a person who is experiencing fewer hypoglycaemic events may be doing so because they are maintaining their glucose levels above target range (9), a practice that confers well-documented health risks (45, 46). Given that dogs are shown to be alerting to hyperglycaemia, categorizing any alerts that occurred outside of hypoglycaemia as “incorrect” would clearly lead to a misleading measure of performance. Our results highlight the importance of considering hyperglycaemic episodes and longer-term HbA1c levels in future when assessing DAD effectiveness.

When performance was calculated using clinical set points for extreme hypoglycaemia (rather than individual ranges) the cohort sensitivity was reduced slightly to 54.2% whereas the PPV to out-of-range episodes reduced from 69.7 to 50.4%. The use of clinical set points has been advocated in previous studies [e.g. (30, 31)] but may not reflect the glucose levels to which the dogs have been trained to respond. Therefore, definitions of hypo- and hyperglycaemia should be considered in future studies assessing DAD performance, especially if the specifications of the dogs' training values are at odds with the values imposed for performance analysis.

Our findings suggest that pre-alerting is perhaps not as common as DAD owners report, but that it does occur, as we find three dogs showing greater sensitivity to lows, and three dogs showing greater sensitivity to highs when we include a 15-min window prior to the first OOR recording. As a cohort, when alerts 15 min prior to the first OOR glucose value are considered incorrect, sensitivity to hypoglycaemic episodes decreases from 55.9 to 51.5%, and hyperglycaemia from 37.3 to 32.9%. Furthermore, when including a ± 0.5 mmol/L margin for the definition of an OOR episode, PPV increases from 69.7 to 76.5%. This suggests that imposing a precise cut-off glucose level may not best represent the DAD's function in alerting to transitioning glucose levels. These comparisons allow us to understand further the effect of methodology on reported performance values and should be considered in future DAD assessment studies.

It is important to consider that no glucose monitoring device will provide identical results to finger-prick blood tests (47). Only two participants reached the Abbott FreeStyle Libre reported 99.7% of readings in zones A and B (35). The FGMS used in this study logs glucose data every 15 min, meaning that readings were compared to the closest temporally to the time of the blood test, which may have affected agreement levels. The agreement for participants Four and Eight is notably below the accepted levels (Figure 3). The relative agreement was however not associated with measured performance of the dogs (Figure 4). Objective studies using a CGMS system that provides a continual glucose trace would be optimal and are still required.

We saw a number of attributes of the partnership that were associated with better performance, which supports Rooney et al.'s (32) findings. Increased sensitivity was linked to *Client's Confidence in the Dog's Ability, Dog's Motivation and Enjoyment of the Task* and *Strength of Dog's Alert*. Increased PPV was associated with *Client's Confidence in the Dog's Ability* and showed a tendency to be higher with increased *Owner's Willingness to Reward Alerts* and *Dog's Motivation and Enjoyment of the Task* (Figure 4). It should be noted that across all analyses we included only unambiguous alerts to ensure a conservative assessment of DAD accuracy. However, there were some instances of ambiguous attention seeking behaviors that were unclear to both coders and seemingly also to owners. This is of interest given that the DAD's instructor rating of *Strength of Dog's Alert* was associated with increased sensitivity and may point toward a greater emphasis on developing non-ambiguous alerts (e.g., fetch blood testing kit) during the training process. These findings add to our current understanding of what makes a successful partnership and which traits in both dog and owner should be targeted during selection and matching, and further developed during the training process.

Similar to Rooney et al. (32) we saw a decrease in sensitivity and PPV in dogs that had been accredited for longer. This suggests that whilst dogs finish their training period responding reliably to OOR episodes, correct owner responses to alerts in the home environment may not be maintained in all dogs. Once placed, in some cases inconsistent rewarding may, with time, reduce the dogs' sensitivity and specificity to hypoglycaemic episodes. Examination of CCTV footage showed variability in owners' adherence to training protocol when responding to a DAD alert. We found that a higher percentage of alerts followed by a blood test, and a lower percentage of ignored alerts, tended to be associated with increased sensitivity and PPV (Figure 4). Participants Three and Five, for example, showed high levels of compliance by testing their blood following 100% of spontaneous DAD alerts, and always testing prior to rewarding (Table 3). Their dogs also showed high levels of sensitivity and PPV within the cohort (Figure 2). In contrast, owners shown to ignore spontaneous DAD alerts were found to have “poorer performing” dogs (Figure 4). Lack of rewarding, as well as rewarding prior to blood testing, are against advised protocol and could lead to the dog become de-trained, since they inadvertently may learn that alerting does not result in a reward. This may begin the process of behavioral extinction, or shape the behavior such that the DAD learns it can gain a reward regardless of the accuracy of their response. While reinforcement training is rigorous during the dog's initial training it is likely that post-accreditation owners vary in their ability to maintain consistent training whilst concurrently managing their diabetes. Incorrect rewarding may occur due to cognitive impairments during glucose fluctuations, or due to owners relaxing their training protocol over time. However, since owners who were observed following training protocol correctly had more successful dogs, this highlights the importance of regular monitoring and continuation training of both dog and owner and the potential value of using CCTV for monitoring. Given the small number of dogs sampled however, this study should be considered as exploratory. The substantial variation seen between these dogs suggests that further investigation is important to fully understanding the mechanisms underlying variation in DAD performance.

## Conclusion

Owner-independent measures demonstrate that trained dogs can alert their owners to both hypo- and hyperglycaemic blood glucose levels, with variable but significant accuracy. We found that using clinical vs. individual glycemic range values did not have a substantial effect on the reported sensitivity rates of DAD but may impact on calculated PPV if imposing glucose ranges to which the dogs had not been trained to respond. DAD accuracy was affected by aspects of data analysis, such as whether 15-min pre-alerting periods were deemed correct or whether we included a ± 0.5 mmol/L margin of error around glucose levels. This indicates that methodological factors of analysis can influence reported DAD accuracy levels and should be considered carefully in future assessments.

Whilst DADs clearly have the ability to detect OOR glucose levels, their success relies not only on the quality of their initial training, but also on post-accreditation factors such as their placement environment and reward systems during their working life. Our findings point toward a need for further prospective investigation into factors predicting successful partnerships and close monitoring of owner and dog behavior in order to maintain performance post-accreditation. This study supports the idea that DADs can function as an important additional tool and component of a diabetes plan to facilitate tightened glycemic control, and should complement developing diabetes technology, rather than replace it. Results presented here could inform strategies to optimize the relationship between owners and their dogs, training programmes, and alerting performance in the future.

## Data Availability

The datasets generated for this study are available on request to the corresponding author.

## Ethics Statement

This study was carried out in accordance with the recommendations of The University of Bristol Faculty Research Ethics Committee (UB/17/014) and The University of Edinburgh Royal (Dick) School of Veterinary Sciences Human Ethics Research Committee with written informed consent from all subjects. All subjects gave written informed consent in accordance with the Declaration of Helsinki. The protocol was approved by The University of Bristol Faculty Research Ethics Committee (UB/17/014) and The University of Edinburgh Royal (Dick) School of Veterinary Sciences Human Ethics Research Committee. This approval covered ethical consideration of human and animal participants.

## Author Contributions

CW carried out the majority of the data collection and video coding, parts of the analysis and jointly drafted the paper. NR designed the study, jointly drafted the manuscript, and will act as guarantor for the content to the paper. SM carried out the main statistical analyses and commented on the draft manuscript. SK carried out video coding for two of the partnerships' CCTV footage and second coded two further participants' footage. CG fed into the study design at numerous stages and commented on the draft manuscript. CP was integral in facilitating data collection, teaching CW how to administer FGMS devices to participants, fed into the study design and helped with interpretation of the data and commented on the final manuscript.

### Conflict of Interest Statement

CW was given a salary by Medical Detection Dogs (MDD) to develop this paper, which formed the basis of her MSc thesis into a journal article. SM, who did the majority of the analyses, has no financial interest in Medical Detection Dogs. CP is a full-time employee of MDD, as Medical Liaison officer. CG is a co-founder of MDD. NR works primarily for The University of Bristol but is also a part-time paid employee of MDD. The remaining author declares that the research was conducted in the absence of any commercial or financial relationships that could be construed as a potential conflict of interest.
